# Cutting Through Time: A Surgical Comparison of Bosworth, LARS™, and TightRope^®^ for AC Joint Dislocations

**DOI:** 10.3390/jfmk10040375

**Published:** 2025-09-29

**Authors:** Domenik Popp, Arastoo Nia, Sara Silvaieh, Cornelia Nass, Stephan Heisinger, Lorenz Pichler, Thomas M. Tiefenboeck

**Affiliations:** 1 Clinical Division of Traumatology, Department of Orthopedics and Trauma Surgery, Medical University of Vienna, 1090 Vienna, Austria; arastoo.nia@meduniwien.ac.at (A.N.);; 2 Department of Orthopedics and Trauma-Surgery, University Hospital Neunkirchen, 2620 Neunkirchen, Austria; 3 Department of Neurology, Medical University of Vienna, 1090 Vienna, Austria

**Keywords:** LARS, tight-rope, Bosworth, ACJ, luxation, Rockwood

## Abstract

**Background:** Acromioclavicular joint (ACJ) injuries frequently result from trauma to the shoulder girdle and are particularly common among young, physically active individuals. These injuries account for approximately 9% of all traumatic shoulder girdle injuries and often lead to functional impairment and pain. The TightRope^®^ system, LARS™ band, and Bosworth screw are among over 160 currently described surgical techniques for managing ACJ dislocations. However, there is no consensus regarding the optimal surgical approach, particularly for the management of moderate Rockwood Type III ACJ dislocations. **Materials and Methods:** In this retrospective study, data from 246 patients who underwent surgery for ACJ dislocation between 2010 and 2018 at the Department of Orthopedics and Trauma Surgery, Medical University of Vienna, were analyzed. Patients were divided into four cohorts based on the surgical technique used: Bosworth screw, LARS (acute), LARS (chronic), and TightRope. Clinical and radiological outcomes were assessed pre- and postoperatively using the Visual Analog Scale (VAS), Constant, Disability of the Arm, Shoulder and Hand Score (DASH), Simple Shoulder Test (SST), University of California—Los Angeles Shoulder Score (UCLA), Short Form Health Survey (SF-36), and American Shoulder and Elbow Surgeons score (ASES), as well as radiographic analysis. Radiological measurements of the acromioclavicular (AC) and coracoclavicular (CC) joint spaces were taken on both the injured and uninjured shoulders to analyze and compare the reduction in joint gaps. **Results:** All surgical methods resulted in significant reductions in AC and CC joint gaps. The TightRope and LARS acute groups showed the greatest reductions, with minimal complication rates. Complication analysis revealed significant differences in clavicular elevation (*p* < 0.001) and CC-ligament ossification (*p* = 0.006), which were most frequent in the Bosworth group and least common in TightRope^®^ patients, with LARS showing intermediate values. AC joint arthrosis was uncommon in all four groups and did not differ significantly (*p* = 0.13). Overall, TightRope^®^ was associated with the most favorable complication profile. The postoperative VAS score in the TightRope group was 1.52 ± 2.06, and the Constant score was 96.83 ± 5.41, reflecting high patient satisfaction. **Conclusions:** All systems led to satisfactory radiological and clinical outcomes, with the LARS™ band showing particular effectiveness in chronic ACJ dislocations. While all techniques provided good results, the TightRope^®^ system demonstrated the most favorable overall profile in our cohort and may therefore be considered a promising contemporary option. Further studies are needed to determine the optimal treatment for moderate ACJ dislocations and to assess the cost-effectiveness of these surgical techniques.

## 1. Introduction

The acromioclavicular (AC) joint is essential for shoulder stability and upper limb function [1]. Acromioclavicular joint injuries are relatively common, accounting for around 9% of shoulder girdle trauma with an incidence of 3–4 per 100,000 individuals [2,3]. These injuries typically result from falls onto an outstretched arm, applying excessive force to the ligamentous structures [4]. The severity can range from mild capsular strain to complete ligament ruptures and joint dislocation. Representing 2–16% of all joint dislocations, AC joint injuries contribute significantly to shoulder pathology [5].

Patients usually present with pain, impaired shoulder function, and pseudo-elevation of the lateral clavicle end [6]. Treatment aims to restore function and prevent long-term morbidity [7]. Management is guided by injury severity and typically classified through clinical examination and imaging [8,9,10].

Acromioclavicular dislocations are most common in young, active individuals, with peak incidence in the second and third decades of life. Males are affected up to five times more, and in athletes, this ratio increases to eight times [11]. Around 25–50% of cases are sports-related. Contact sports carry the highest risk, but injuries also occur during jogging, skiing, biking, motorcycling, or even household accidents [12,13].

Low-grade injuries (Rockwood I–II) are usually treated conservatively with immobilization and analgesia, and outcomes are generally good [14,15,16]. However, Type III injuries remain controversial, with no clear consensus on the necessity of surgery [1,16]. Treatment often depends on the patient’s activity level and functional demands. Despite favorable outcomes with conservative therapy, retrospective studies report long-term complications such as persistent pain and limited range of motion in some cases [17,18]. Consequently, delayed surgical intervention becomes necessary in 10–27% of patients, although chronicity and fibrotic tissue may limit surgical success. Fibrotic tissue can compromise surgical outcomes by altering normal tissue architecture, reducing elasticity, and promoting adhesions that restrict repaired structures. Its poor vascularity and cellularity further impair tendon-to-bone healing, collectively increasing the risk of stiffness, pain, and incomplete functional recovery [17,19].

For high-grade injuries (Rockwood IV–VI), surgical treatment is standard [4,9,20,21,22]. Numerous techniques have been developed, including the use of synthetic ligament systems and minimally invasive, arthroscopically assisted procedures aimed at improving recovery and shortening rehabilitation time [23]. Older methods using metal plates or screws are declining in use due to joint stiffness and the need for complex postoperative care. However, newer approaches still require evaluation regarding their long-term effectiveness and ability to replicate native ligament function [9]

The broad array of surgical techniques in the literature reflects the lack of a standardized approach [22,24,25]. Over the past decades, methods have evolved considerably [26]. In the 1980s, transfixation with K-wires and hook plates was gradually replaced by the Bosworth screw, which focused on restoring the coracoclavicular distance to promote natural healing [27]. However, the need for hardware removal and limited postoperative mobility prompted the development of dynamic fixation systems such as the Ligament Augmentation Reconstruction System (LARS™) [28].

The shift toward minimally invasive surgery has further influenced AC joint procedures. The arthroscopically implantable TightRope^®^ system has become widely used due to advantages like bi-axial stability and faster recovery [29,30]. Despite progress, some older procedures remain in use, though their role is diminishing. Treatment choice often depends on institutional protocols and surgeon preference, with variability even within the same department, emphasizing the lack of a universally accepted standard [31].

To address these uncertainties, this study evaluates the radiological and clinical outcomes of three established surgical techniques for AC joint dislocations.

## 2. Materials and Methods

### 2.1. Study Design and Patient Selection

This retrospective cohort study was conducted at the Department of Orthopaedics and Trauma Surgery, Division of Trauma Surgery, Medical University of Vienna. It included patients who underwent surgical treatment for acromioclavicular (AC) joint dislocation between January 2003 and December 2018, using the TightRope^®^ system, LARS™ ligament, or Bosworth screw technique.

Inclusion criteria were a radiologically confirmed AC joint dislocation of Rockwood Type III or higher, surgical treatment using one of the above techniques and written consent to participate in this study. Patients were excluded if they had prior injuries, instability, or surgery involving the affected shoulder, or additional trauma to the shoulder complex. A total of 246 patients met the criteria and were grouped according to the surgical method: TightRope^®^ (*n* = 65), LARS™ for acute (*n* = 110) or chronic cases (*n* = 42), and Bosworth screw (*n* = 29). Chronic cases were defined as those treated surgically more than 30 days post-injury with persistent symptoms after failed conservative management.

Surgical indication was based on clinical presentation (pain, piano key-sign, limited ROM) and radiological findings.

### 2.2. Radiological Assessment

**Figure 1:**
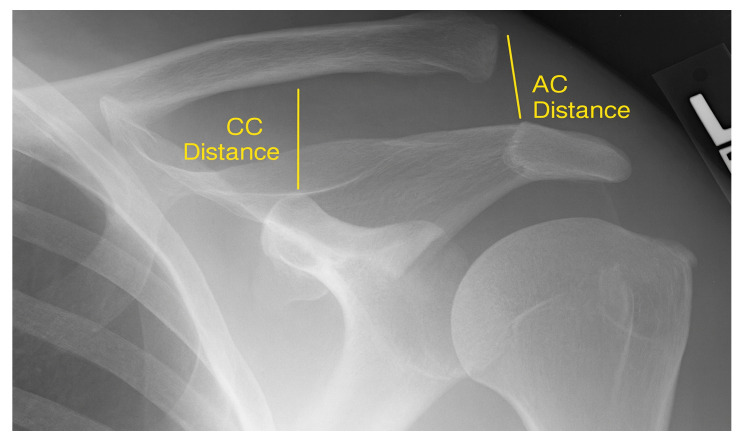
Anteroposterior radiograph of the shoulder demonstrating measurement of the acromioclavicular (AC) distance and the coracoclavicular (CC) distance.

Standardized bilateral shoulder radiographs (anteroposterior, axial, and stress views) were obtained pre- and postoperatively. Stress images were taken with 5–10 kg weights in each hand. The primary radiographic parameters were the acromioclavicular (AC) distance—measured from the inferior acromion to the clavicle—and the coracoclavicular (CC) distance—from the superior coracoid process to the clavicle (Figure 1). Measurements were also taken from the contralateral shoulder for intra-individual comparison.

All measurements were analyzed by two independent observers using data from the institutional systems (AKIM, KIS) or study-specific follow-up imaging when required.

### 2.3. Clinical Outcome Measures

To evaluate functional outcomes and health-related quality of life, a comprehensive set of validated clinical scoring instruments was employed at the final follow-up.

Pain intensity was assessed using the Visual Analog Scale (VAS) [32].

Functional evaluation of the shoulder was performed using the Constant Score [33], which integrates both subjective and objective components. The score encompasses four domains: pain (15 points), activities of daily living (20 points), range of motion (40 points), and strength (25 points), resulting in a maximum score of 100. This tool is especially relevant for surgical outcomes due to its balanced representation of clinical function.

The Disabilities of the Arm, Shoulder and Hand (DASH) [34] questionnaire was used to assess upper extremity function and patient-perceived disability. It consists of 30 items rated on a 5-point Likert scale, covering tasks such as dressing, lifting, and performing work or leisure activities. The DASH score is expressed as a percentage, with higher scores indicating greater disability.

The Simple Shoulder Test (SST) [35] provided a binary, easy-to-administer assessment. It consists of 12 yes/no questions targeting specific functional capacities of the shoulder, such as the ability to lift weight overhead or sleep comfortably on the affected side.

Shoulder-specific function was further assessed with the American Shoulder and Elbow Surgeons (ASES) Score [36], which combines a pain scale and a 10-item functional questionnaire to evaluate limitations in everyday tasks. The total score ranges from 0 to 100, with higher values reflecting better shoulder performance.

The UCLA Shoulder Score [37], developed by the University of California, Los Angeles, was also employed to provide a multifactorial assessment. It integrates components for pain, function, active forward flexion, strength, and patient satisfaction, yielding a maximum of 35 points.

To capture broader health-related quality of life, the 36-Item Short Form Health Survey (SF-36) [38] was administered. This generic instrument evaluates eight domains: physical functioning, role limitations due to physical health, bodily pain, general health, vitality, social functioning, emotional role functioning, and mental health. The SF-36 enables a holistic view of the patient’s overall well-being and the systemic impact of shoulder pathology.

### 2.4. Surgical Procedure

#### 2.4.1. Bosworth Screw

Reconstruction of the acromioclavicular (AC) joint was performed according to the Bosworth technique [39]. Patients were placed in the beach-chair position, and fluoroscopy was used to confirm joint reduction and guide implant placement. Through a 2–3 cm mini-open incision medial to the AC joint, the joint was reduced and temporarily stabilized with two parallel K-wires introduced from the acromion into the clavicle. A screw channel was then drilled from the lateral clavicle into the coracoid, tapped to 6.5 mm, and fixed with a partially threaded cortical screw of appropriate length (DePuy Synthes, Oberdorf, Switzerland).

#### 2.4.2. LARS™

Operations were performed under general anesthesia with the patient in the beach-chair position. Through a sagittal incision over the AC joint, the deltoid and trapezius were detached from the lateral clavicle, the joint was exposed, and fibrotic tissue debrided. If necessary, the lateral clavicle was resected to allow anatomical reduction. A LARS ligament was passed around the coracoid base using a guide wire, and two clavicular drill holes were prepared: a 4 mm craniodorsal–caudoventral hole lateral to the coracoid and a 4.5 mm cranioventral–caudodorsal hole medial to it. The ligament was first fixated in the lateral tunnel with an interference screw, tensioned to achieve reduction, and then secured medially with a second interference screw. Reduction and fixation were confirmed fluoroscopically, and the deltoid was reattached before layered closure [40].

#### 2.4.3. TightRope^®^

Arthroscopic TightRope^®^ fixation was performed under general anesthesia with the patient in the beach-chair position. After confirming reducibility, a 1 cm incision was made over the lateral clavicle, the deltotrapezial fascia was incised, and the AC joint temporarily reduced with a K-wire under fluoroscopic control. Diagnostic arthroscopy was performed via the posterior portal, with anterolateral and accessory anterolateral portals established to access the coracoid base, which was exposed using radiofrequency. A C-guide for ACJ reconstruction (Arthrex, Naples, FL, USA) was positioned, and a 4 mm tunnel was drilled from clavicle to coracoid. A Nitinol wire was used to shuttle the TightRope^®^ device, seating the button beneath the coracoid, after which reduction was confirmed fluoroscopically and the clavicular washer secured with tied sutures. The deltotrapezial fascia and skin were closed in layers [41].

### 2.5. Study Objectives and Statistical Analysis

The study aimed to compare clinical and radiological outcomes of three surgical techniques: TightRope^®^ (Arthrex, Naples, FL, USA), LARS™ (Laboratoire d’Anatomie, Lyon, France), and Bosworth screw. While conservative therapy is well established for low-grade injuries, optimal management of higher-grade AC dislocations remains debated. The study also evaluated the role of LARS™ in chronic cases following failed conservative treatment.

The primary outcome was the change in AC and CC distances from preoperative assessment to final follow-up (“Delta AC” and “Delta CC”) across the four groups.

Secondary analysis compared changes within each cohort relative to the contralateral shoulder. Additionally, patient-reported outcome scores were compared across cohorts.

Descriptive statistics were calculated for each cohort. Continuous variables were summarized using mean, standard deviation, quartiles, median, and range. Categorical variables were reported as frequencies and percentages. For non-normally distributed data, medians and 95% confidence intervals were provided. Data were visualized using boxplots, histograms, bar and strip charts. Unpaired t-tests were used for continuous variable comparisons. A *p*-value < 0.05 was considered statistically significant. Statistical power was set at 0.80. Analyses were conducted using Microsoft Excel and SPSS Statistics 29 (IBM Corp., Chicago, IL, USA).

Sample size was estimated based on an alpha of 0.05, beta of 0.20 (power = 80%), medium effect size (f = 0.3), and equal allocation across four groups. Using standard ANOVA-based formulas, the required sample size was approximately 132 patients (33 per group). With 246 patients enrolled, the study was adequately powered to detect clinically relevant differences between treatment strategies.

## 3. Results

### 3.1. Epidemiological Data

The descriptive analysis includes patients treated across four operative cohorts: “Bosworth,” “LARS acute,” “LARS chronic,” and “TightRope.” Mean patient age varied slightly among groups, with the Bosworth cohort comprising the oldest patients (mean 38.3 ± 15.53 years). The “LARS acute” and “LARS chronic” cohorts had mean ages of 35.64 ± 11.33 and 34.69 ± 11.46 years, respectively. The TightRope group exhibited the youngest mean age at 34.63 ± 11.83 years. There were no significant differences in patient age between the surgical groups (*p* = 0.88) (Table 1).

All cohorts demonstrated a pronounced male predominance. The proportion of male patients was 94% in both the “LARS acute” (*n* = 103) and TightRope (*n* = 61) cohorts, 90% in the Bosworth group (*n* = 26), and 86% in the “LARS chronic” group (*n* = 36).

Injury severity, classified according to the Rockwood system, revealed a predominance of Rockwood Type III injuries across all cohorts: 62% in both the Bosworth and “LARS chronic” groups, and 58% in the TightRope cohort. Higher-grade injuries (Rockwood Types IV and V) were more prevalent in the “LARS acute” (48%) and TightRope (37%) cohorts (Table 1). There were no statistically significant differences in Rockwood classification between the surgical groups, including for Rockwood type III (*p* = 0.11).

Regarding trauma mechanisms underlying acromioclavicular joint (ACJ) dislocations, sports-related injuries accounted for the highest number of cases (*n* = 93; 37.8%). Other frequent causes included bicycle accidents (*n* = 60; 24.4%), falls (*n* = 56; 22.8%), and motorcycle accidents (*n* = 37; 15.0%).

The mean time from injury to surgical intervention varied significantly across cohorts (Table 1) (*p* = 0.004). In the Bosworth group, the average interval was 26.97 days (SD ± 65.52), with a range from 0 to 294 days. The LARS acute cohort exhibited the shortest interval, with a mean of 10.88 days (SD ± 6.2), while the LARS chronic cohort demonstrated a considerably prolonged interval, averaging 62.43 days (SD ± 34.55). In the TightRope^®^ cohort, the mean time from injury to surgery was 11.14 days (SD ± 7.15).

### 3.2. Clinical Outcomes

At final follow-up, clinical outcomes were assessed using validated patient-reported outcome measures (PROMs) (Table 2). The VAS for pain revealed the most favorable result in the LARS acute cohort, with a mean score of 1.48 (SD ± 2.00), indicating minimal residual pain. In contrast, the Bosworth group demonstrated the highest pain levels, with a mean VAS score of 3.33 (SD ± 1.25). However, across all groups, functional outcome scores (Constant, DASH, ASES, SST, and UCLA) were high and did not differ significantly. Pain levels (VAS) were significantly higher in the Bosworth group compared with the other techniques (*p* < 0.001).

The highest functional outcome was observed in the TightRope^®^ cohort, which achieved a mean Constant Score of 96.83 (SD ± 5.41). Slightly lower values were recorded in the Bosworth and LARS acute cohorts, with mean scores of 92.54 (SD ± 19.82) and 91.36 (SD ± 16.45), respectively. Both groups also exhibited greater variability in functional outcomes compared to the TightRope^®^ cohort.

A similar pattern was seen in the DASH-Score. The TightRope^®^ (mean 4.87, SD ± 9.60) and LARS acute (mean 5.40, SD ± 11.23) cohorts achieved the most favorable outcomes, reflecting minimal disability. The Bosworth group again showed comparatively inferior results (mean 9.26, SD ± 19.39) with a wider distribution, indicating greater heterogeneity in patient-reported disability.

Quality-of-life assessment with the SF-36 revealed differences in selected subdomains: patients treated with LARS acute scored significantly higher in general health (*p* < 0.001) and vitality (*p* = 0.002) compared with Bosworth and TightRope. Other SF-36 domains, including role physical, social functioning, role emotional, and mental health, showed no significant differences between groups.

### 3.3. Radiological Outcome

Comparison of pre- and postoperative radiological parameters, specifically the acromioclavicular (ΔAC) and coracoclavicular (ΔCC) distances, showed no statistically significant differences between the four surgical cohorts, as determined by a Type III F-test (*p* = 0.138 for ΔAC, *p* = 0.623 for ΔCC). Accordingly, no post hoc significance testing was performed, and Table 3 and Table 4 do not report *p*-values from direct group comparisons.

Preoperatively, the mean coracoclavicular distance (CC_pre) was highest in the TightRope group (1.8983 cm ± 0.6267) and lowest in the Bosworth cohort (1.6514 cm ± 0.5538). Under stress imaging (Weight Bearing/WB) (CC_pre_WB), the highest value was observed in the LARS chronic group (2.0838 cm ± 0.4281), while TightRope recorded the lowest (1.9312 cm ± 0.7139).

Postoperative imaging demonstrated a significant reduction in absolute CC distance across all cohorts. The LARS chronic group retained the highest final CC measurement (CC_final: 1.3981 cm ± 0.3677), while Bosworth had the lowest (1.1471 cm ± 0.3550). This pattern was mirrored in stress imaging (CC_final_WB), with LARS chronic again showing the highest postoperative value (1.5662 cm ± 0.3732) and Bosworth the lowest (1.2786 cm ± 0.3182).

For the AC distance, the TightRope (1.4489 cm ± 0.6159) and LARS chronic (1.1362 cm ± 0.5061) cohorts had the highest preoperative values (AC_pre). In contrast, Bosworth and LARS acute reported lower preoperative means (0.7884 cm ± 0.3068 and 0.9489 cm ± 0.4171, respectively). Postoperatively, all cohorts showed reductions in AC distance, with TightRope maintaining the highest mean (AC_final: 0.7289 cm ± 0.4853) and Bosworth the lowest (0.5282 cm ± 0.2597). Under stress (AC_final_WB), the trend persisted: TightRope showed the highest postoperative value (0.8641 cm ± 0.5276), and LARS accrued the lowest (0.6520 cm ± 0.3022).

Overall, all surgical techniques led to substantial postoperative reductions in AC and CC distances. Notably, LARS chronic and TightRope demonstrated the most pronounced radiological improvements. Radiographic evaluation of AC and CC distances is presented in Table 3 and Table 4. No statistically significant differences were observed between the surgical techniques for any of these measurements.

Comparison of acromioclavicular (ΔAC) and coracoclavicular (ΔCC) distances before (pre) and after (final) surgery revealed a significant reduction in joint space on the injured side relative to the uninjured shoulder within each cohort. This indicates a substantial postoperative correction in joint alignment. Among all groups, the LARS acute and TightRope cohorts demonstrated particularly strong effects, with highly significant results (both *p* < 0.0001). Full statistical outcomes are presented in Table 5.

### 3.4. Complications

At the time of final follow-up, radiological assessments were also conducted to evaluate the incidence of long-term complications. These included secondary clavicular elevation, acromioclavicular (AC) joint osteoarthritis, and postoperative ossifications. The frequencies of these complications are summarized in Table 6.

Analysis of radiographic findings demonstrated significant differences in clavicular elevation between the surgical groups (*p* < 0.001), with absence of elevation most frequently observed in the TightRope^®^ group, whereas marked elevation was more common after Bosworth fixation. Patients in the Bosworth group showed higher rates of marked elevation, with 6.9% presenting a double shaft width and 20.7% a single shaft width, compared with 0% and 9.2%, respectively, in the TightRope^®^ group. The LARS groups showed intermediate values, with 10.0% (acute) and 26.2% (chronic) displaying a single shaft width, and 29.1% (acute) and 21.4% (chronic) showing a half shaft width. In contrast, the majority of TightRope^®^ patients (70.8%) exhibited no elevation, whereas this was less frequent in the Bosworth cohort (31.0%).

The prevalence of CC ligament ossification also varied significantly between groups (*p* = 0.006), with Fisher’s exact test confirming a higher risk in Bosworth compared with TightRope^®^ (*p* = 0.0007). The highest prevalence was observed in the Bosworth group (37.9%), followed by LARS acute (21.8%) and LARS chronic (19.1%), while ossification was least common in the TightRope^®^ group (7.7%) However, trends showing increased ossification in Bosworth versus LARS groups did not reach significance.

By comparison, the occurrence of AC joint arthrosis was low across all cohorts and did not differ significantly (*p* = 0.13). The prevalence ranged from 17.2% in the Bosworth group to only 3.1% in the TightRope^®^ cohort, pairwise testing revealed a higher prevalence in Bosworth compared with TightRope^®^ (*p* = 0.027). Both LARS groups showed similar low frequencies (8.2% acute, 7.1% chronic). Overall, these findings suggest that clavicular elevation and CC-ligament ossification were more common in the Bosworth group, whereas TightRope^®^ patients demonstrated the most favorable radiographic profiles.

## 4. Discussion

In this study, all surgical techniques for acromioclavicular joint reconstruction achieved satisfactory clinical and radiological outcomes. Among the methods evaluated, TightRope^®^ fixation provided the most consistent functional results with low variability, while synthetic ligament reconstructions (LARS™) also performed well and demonstrated outcomes comparable to or better than Bosworth fixation. Bosworth fixation, although yielding acceptable results, was associated with higher rates of ossification and less consistent functional scores. Overall, the findings highlight the reliability of modern synthetic ligament techniques and TightRope^®^ fixation in the management of AC joint dislocations.

Despite decades of experience, no consensus exists on optimal surgical treatment. Over 160 techniques have been described [42], with uncertainty persisting, especially for Rockwood type III injuries [15,25]. Recently, a clear trend has emerged toward minimally invasive approaches, even for traumatic cases [26]. Surgical emphasis has shifted to anatomic ligament reconstruction for restoring biomechanics and joint stability [27]. Spencer et al. [43] demonstrated the biomechanical benefits of such reconstructions, which support improved outcomes.

Synthetic fiber-based implants now predominate due to favorable biomechanical properties, biocompatibility, durability, and lower infection risk, replacing many rigid fixation methods [9,25,44]. Advances in materials science continue to influence broader surgical innovation.

Consequently, standalone Bosworth screws and LARS™ reconstructions are declining for acute, high-grade injuries. Current best practices increasingly favor minimally invasive TightRope^®^ fixation or biologically augmented hybrid techniques for chronic cases after failed conservative therapy [15].

Clinical and radiological outcomes in this study were generally satisfactory across all surgical techniques, including Bosworth screw, as well as synthetic ligament augmentation with LARS™ and TightRope^®^ systems.

The Bosworth group demonstrated good to very good outcomes in physical function, social activity, and pain control based on patient-reported measures (see Table 2). However, the wide Constant score variability (mean 92.54 ± 19.82) suggests ongoing functional complaints in some patients. In contrast, the TightRope^®^ group achieved the highest Constant score (mean 96.83 ± 5.41) with the least variability, indicating more consistent and effective results.

Compared with historical data, Bosworth outcomes appear improved. Broos et al. [45] reported implant failure in 16%, calcification in 39%, arthritis in 41%, and only 60% favorable results. In this study the Bosworth cohort (*n* = 29) had lower dissatisfaction (10%), calcification (37.9%), and arthrosis (17.2%) at final follow-up.

Re-dislocation rates also declined over time—from 25% in Broos et al. [45] to 11.76% in Darabos et al. [9]. These improvements may reflect advances in surgical technique, perioperative care, and rehabilitation. However, interpretation remains limited by small cohort sizes and methodological differences across studies.

The “LARS acute” cohort showed favorable outcomes in recovery and functional restoration. Short Form-36 Health Survey results indicated high functionality and quality of life. This group achieved the highest SF-Vitality score (mean 73 ± 13.84), outperforming the TightRope^®^ group by 10.31 points (mean 62.69 ± 17.16), which had the lowest value in this domain.

Pain control was satisfactory, with a mean VAS of 1.48 (±2.00) and a Constant score of 91.36 (±16.45). However, the large standard deviation indicates considerable interindividual variability. Despite a high mean Constant score, this cohort had the lowest average functional performance among all groups. Still, 93.1% of patients reported being “fully” or “mostly” satisfied with the outcome.

In comparison, Lu et al. [22] reported a mean Constant score of 94.5 (±9.3) and VAS of 0.7 (±1.4) at 3 years in 24 patients treated with LARS™, with a low complication rate. The study cohort had a higher rate of calcification (21.8%) versus Lu’s (16.7%) [22].

Marcheggiani Muccioli et al. [40] also noted low complication rates and only 2% re-dislocation at 24 months, supporting long-term efficacy. Trieb et al. [46] confirmed histological biocompatibility of LARS™, while Liu et al. [47] reported just 1.5% elongation under 1700 N tensile load, demonstrating excellent mechanical durability.

Despite this mid-term period, Tiefenboeck et al. [48] showed sustained excellent-to-good outcomes up to 7.4 years after LARS™ augmentation.

The TightRope^®^ cohort (*n* = 65) demonstrated consistently good to excellent outcomes across physical and psychosocial domains. Pain scores were low (VAS 1.52 ± 2.06), and functional scores were among the highest, including a Constant score of 96.83 (±5.41) and DASH score of 4.87 (±9.6). The DASH score was the most favorable among all acute AC dislocation groups, outperforming both the Bosworth (9.26 ± 19.39) and LARS acute (5.4 ± 11.23) cohorts, with lower variability. These findings support successful functional restoration and high patient satisfaction.

Subjective evaluation confirmed this, with 89.2% reporting they were “fully” or “mostly” satisfied. These outcomes align with literature. Nie et al. [30] showed TightRope^®^ outperformed hook plates in VAS (1.2 ± 0.6 vs. 1.8 ± 1.1) and Constant scores (89.3 ± 4.2 vs. 83.3 ± 8.8). Jensen et al. [49] similarly reported median VAS of 0.4 with TightRope^®^ versus 0.8 for hook plates in a cohort of 56 patients.

Postoperative complication rates were low. At a mean 86.4 months follow-up, only 3.1% showed osteoarthritis and 7.7% had calcifications. In a separate study of 29 patients, Dobbelaere et al. [50] also reported excellent mid-term outcomes with mini-open TightRope^®^ fixation.

The literature provides limited guidance on surgical indications for chronic AC dislocations and remains a challenging clinical problem, with numerous surgical techniques proposed to restore stability and function [15]. Literature about this specific topic is scarce. Among the techniques evaluated, only the LARS™ ligament is explicitly approved as a stand-alone option for chronic cases. Dislocations persisting beyond 4 weeks are typically classified as chronic, and conservative treatment is usually attempted first, though it often fails to achieve adequate symptom relief or functional recovery [51].

Surgical repair in chronic cases often uses autologous tendon grafts rather than synthetics [5,52,53]. Though LARS™ is formally approved for this use, it remains uncommon in routine practice. Fauci et al. [54] reported significantly better outcomes with autologous grafts compared to LARS™ at 4 years, with Constant scores of 94.2 (±4.9) vs. 85.9 (±16), and UCLA scores of 18.2 (±1.7) vs. 15.4 (±4.2). However, surgery was performed earlier in the autologous group (12 vs. 16 months post-injury), following at least 6 months of failed conservative therapy.

Kumar et al. [55] compared subjective outcomes of the modified Weaver-Dunn procedure with synthetic ligament reconstruction in 55 patients with chronic Rockwood grade III–V AC dislocations. At a mean follow-up of 40 months, both groups showed significant improvement in Oxford Shoulder and Nottingham Clavicle Scores, but the synthetic ligament group achieved significantly higher postoperative scores (*p* = 0.008 and *p* = 0.007, respectively). Patients treated with synthetic ligaments also returned to work and sports significantly earlier (6 vs. 14 weeks and 12 vs. 25 weeks; both *p* < 0.001). Persistent pain or recurrent dislocation occurred in three WD patients and one synthetic ligament patient. These findings suggest that synthetic ligament reconstruction provides superior functional recovery and faster return to activity compared with the modified Weaver–Dunn procedure.

Chang et al. [56] conducted a meta-analysis of four trials including Rockwood type III–VI acromioclavicular dislocations to compare CC reconstruction with the modified WD procedure. The pooled data showed that CC reconstruction patients achieved significantly higher postoperative ASES scores (*p* < 0.001), Oxford Shoulder Scores (OSS) (*p* = 0.020), and Nottingham Clavicle Scores (NCS) (*p* < 0.001) than WD, while no significant difference was found in Constant-Scores (*p* = 0.100). Radiographically, CC reconstruction maintained a smaller CC distance under a 10 kg load (*p* < 0.001), indicating greater stability. These results demonstrate that CC reconstruction provides superior functional and radiographic outcomes in chronic cases compared with WD, in line with the clinical findings of Kumar et al. [55], who also reported better functional recovery and earlier return to activity with synthetic ligament reconstruction.

Saccomanno et al. [57] reported good results in 30 patients treated at 28.1 months (±2.4) post-injury (Constant: 91.7 ± 6.6, DASH: 8.2 ± 6.7). In contrast, our LARS™ cohort underwent surgery at a mean of 62 days (±54.55), with follow-up at 8.1 years, achieving the best outcomes among all groups (Constant: 95.52 ± 5.45; DASH: 4.66 ± 6.94). All had failed prior conservative treatment.

These findings support early surgical intervention, even in chronic cases, especially given evidence that full biological healing is limited to the early post-traumatic phase [17,19,58]. Radiologically, all four techniques significantly reduced AC and CC joint distances, with no statistically significant differences between groups. The LARS chronic cohort showed favorable delta values, suggesting stabilization not achieved with conservative care. Overall, all methods effectively restored joint alignment.

Although Rockwood type IV injuries were more common in the “LARS acute” (25.5%) and “TightRope” (26.2%) groups than in “LARS chronic” (19%), joint reduction remained effective. Mean deltas were 0.477 cm (±0.38) for AC and 0.275 cm (±0.513) for CC distances.

Considering cohort sizes and the composition of patient samples, the data suggest a temporal trend toward increased adoption of synthetic ligament techniques—namely the LARS™ ligament and TightRope^®^ system—while the Bosworth screw has seen a decline in clinical use over time.

### Limitations

The relatively small and uneven cohort sizes, particularly in the Bosworth group, limit the generalizability of these findings. The retrospective design and no conservative control group also introduce methodological limitations, including lack of randomization and standardized follow-up. Prospective studies with larger, balanced cohorts and longer follow-up are needed to validate these results and assess long-term stability of the examined techniques.

Clinical evaluations were conducted only once postoperatively, with no baseline preoperative scores available, making it impossible to quantify functional improvement. Reliance on patient-reported outcomes may introduce recall bias, misclassification of symptoms, and difficulty establishing causal relationships.

Data quality presents another limitation, as some information was extracted from institutional databases rather than collected directly. Given the retrospective nature, certain clinical inputs may lack the precision or context required for robust scientific interpretation.

## 5. Conclusions

This study demonstrates that all evaluated surgical techniques—including those applied in chronic AC joint dislocations—resulted in significant radiographic reduction in AC and CC distances relative to the uninjured side, along with favorable patient reported outcomes. The “LARS acute” and “TightRope” cohorts showed particularly effective postoperative reduction and low complication rates, although differences between cohorts were not statistically significant.

The TightRope^®^ system demonstrated favorable outcomes in acute, high-grade dislocations, while LARS™ reconstruction without biological grafts proved to be a feasible option for chronic cases after failed conservative management.

Further prospective studies with longer follow-up are needed to validate these outcomes and determine optimal strategies, especially for moderate-grade injuries where consensus is lacking.

## Figures and Tables

**Table 1:** Descriptive statistics of the patient cohort.

Variable	Bosworth	LARS Acute	LARS Chronic	TightRope
**age (years)**				
mean	38.3	35.64	34.69	34.63
SD	15.53	11.33	11.46	11.83
median	33.48	33.75	31.2	29.89
1st quartile	25.87	27.41	27.93	25.92
3rd quartile	50.1	40.68	37.34	42.47
minimum	16.13	17.22	20.11	16.64
maximum	75.76	69.64	69.4	61.04
**Rockwood Classification**				
Rockwood III	23 (79%)	57 (52%)	26 (62%)	41 (63%)
Rockwood IV	4 (14%)	28 (25%)	8 (19%)	17 (26%)
Rockwood V	2 (7%)	25 (23%)	8 (19%)	7 (11%)
**time to surgery (days)**				
mean	26.97	10.88	62.43	11.14
SD	65.52	6.2	34.55	7.15
median	9	10	37	10
1st quartile	4	6	34.25	6
3rd quartile	18	15	93.25	13
minimum	0	0	30	0
maximum	294	25	189	42
**operating time (minutes)**				
mean	77.41	70.73	72.71	86.48
SD	24.6	21.38	23.81	34.5
median	75	70	65	85
1st quartile	55	55	55	60
3rd quartile	95	80	75	105
minimum	40	40	50	20
maximum	135	150	140	170
**follow-up (months)**				
mean	196.9	97.5	88.1	86.4
SD	55.6	10.5	11.3	12.2
median	192.5	98.6	92.6	90.3
1st quartile	175.9	90.4	80.5	77.9
3rd quartile	230	104.6	95.7	94.1
minimum	160.1	51.4	49.5	66.3
maximum	266.7	181.1	182.4	108.7

SD: Standard Deviation; LARS: Ligament Augmentation Reconstruction System.

**Table 2:** Overview of all scores from clinical follow-up and questionnaires by cohort.

Score	Bosworth (Mean ± SD)	LARS Acute (Mean ± SD)	LARS Chronic (Mean ± SD)	TightRope (Mean ± SD)	*p*-Value
**VAS**	3.33 ± 1.25	1.48 ± 2.00	1.62 ± 2.18	1.52 ± 2.06	**0.0003**
**Constant**	92.54 ± 19.82	91.36 ± 16.45	95.52 ± 5.45	96.83 ± 5.41	0.0853
**DASH**	9.26 ± 19.39	5.40 ± 11.23	4.66 ± 6.94	4.87 ± 9.60	0.7936
**ASES**	93.26 ± 8.34	92.43 ± 13.50	95.39 ± 7.00	94.11 ± 11.81	0.0610
**SST**	96.49 ± 12.82	95.79 ± 9.18	96.21 ± 7.42	95.77 ± 14.37	0.4804
**UCLA**	35.32 ± 13.35	33.59 ± 7.21	33.27 ± 2.84	33.40 ± 4.57	0.3652
**SF-PF**	97.50 ± 4.81	95.43 ± 8.24	94.54 ± 8.18	92.85 ± 11.52	0.0481
**SF-RP**	96.43 ± 8.91	96.18 ± 17.20	96.02 ± 11.85	95.00 ± 17.79	0.8123
**SF-BP**	94.79 ± 14.31	93.75 ± 11.64	88.66 ± 17.91	93.82 ± 11.45	0.0317
**SF-GH**	79.68 ± 17.75	90.28 ± 10.92	86.89 ± 13.75	78.15 ± 13.73	**0.0002**
**SF-VT**	71.43 ± 12.01	73.00 ± 13.84	65.68 ± 16.74	62.69 ± 17.16	**0.0021**
**SF-SF**	97.77 ± 4.88	99.14 ± 5.61	97.73 ± 10.41	95.38 ± 12.80	0.0527
**SF-RE**	94.95 ± 17.48	94.95 ± 20.95	89.82 ± 26.58	96.41 ± 13.34	0.2150
**SF-MH**	85.63 ± 11.16	87.35 ± 9.64	85.10 ± 11.67	84.98 ± 8.49	0.4336

SD: Standard Deviation; LARS: Ligament Augmentation Reconstruction System; VAS: Visual Analog Scale; DASH: Disabilities of the Arm, Shoulder and Hand; SST: Simple Shoulder Test; ASES: American Shoulder and Elbow Surgeons; UCLA Shoulder Score: University of California—Los Angeles Shoulder Score; SF-36: Short Form Health Survey; PF: Physical; RP: Role Physical; BP: Bodily Pain; GH: General Health; VT: Vitality; SF: Social Functioning; RE: Role Emotional; MH: Mental Health.

**Table 3:** Mean CC-distances before surgery and at final follow-up for all four cohorts.

Measurement (cm)	Mean	SD	Median	Q1	Q3	Min	Max
**Bosworth**							
**CC_final**	1.1471	0.3550	1.05	0.905	1.2825	0.70	1.98
**CC_pre**	1.6514	0.5538	1.59	1.24	1.95	0.84	2.77
**CC_final_WB**	1.2786	0.3182	1.21	1.07	1.3975	0.87	2.02
**CC_pre_WB**	2.0183	0.5630	2.08	1.48	2.51	1.12	2.88
**CC_final_uninjured**	1.0982	0.1788	1.05	0.99	1.22	0.76	1.53
**CC_pre_uninjured**	1.1424	0.1957	1.11	1.01	1.30	0.69	1.44
**LARS acute**							
**CC_final**	1.2165	0.3217	1.18	1.00	1.37	0.70	2.73
**CC_pre**	1.7991	0.4466	1.75	1.45	2.07	0.98	2.87
**CC_final_WB**	1.4046	0.3334	1.37	1.14	1.57	0.82	2.83
**CC_pre_WB**	2.0612	0.4798	2.025	1.68	2.42	1.17	3.29
**CC_final_uninjured**	1.1130	0.2048	1.10	0.99	1.22	0.75	1.87
**CC_pre_uninjured**	1.1379	0.1960	1.12	1.00	1.24	0.77	2.21
**LARS chronic**							
**CC_final**	1.3981	0.3677	1.355	1.175	1.435	0.93	2.73
**CC_pre**	1.6733	0.3983	1.58	1.27	1.92	1.18	2.33
**CC_final_WB**	1.5662	0.3732	1.49	1.35	1.7175	1.10	2.83
**CC_pre_WB**	2.0838	0.4281	1.87	1.77	2.42	1.54	3.29
**CC_final_uninjured**	1.1257	0.1539	1.13	1.01	1.20	0.84	1.45
**CC_pre_uninjured**	1.1326	0.1652	1.11	1.00	1.19	0.84	1.53
**TightRope^®^**							
**CC_final**	1.2646	0.3838	1.18	1.02	1.49	0.51	2.09
**CC_pre**	1.8983	0.6267	1.815	1.445	2.305	0.201	3.59
**CC_final_WB**	1.4153	0.4662	1.355	1.0925	1.685	0.57	2.48
**CC_pre_WB**	1.9312	0.7139	1.86	1.73	2.235	0.169	3.38
**CC_final_uninjured**	1.1490	0.2429	1.14	0.96	1.31	0.69	1.73
**CC_pre_uninjured**	1.0409	0.3405	1.04	0.85	1.25	0.11	2.21

SD: Standard Deviation; Q: Quartile; Min: Minimum; Max: Maximum; CC: coracoclavicular; WB: Weight Bearing; LARS: Ligament Augmentation Reconstruction System.

**Table 4:** Mean AC-distances before surgery and at final follow-up for all four cohorts.

Measurement (cm)	Mean	SD	Median	Q1	Q3	Min	Max
**Bosworth**							
**AC_final**	0.5282	0.2597	0.485	0.3275	0.645	0.12	1.23
**AC_pre**	0.7884	0.3068	0.765	0.53	0.99	0.22	1.36
**AC_final_WB**	0.7568	0.3703	0.65	0.5075	0.955	0.36	1.89
**AC_pre_WB**	1.0228	0.2887	1.01	0.83	1.25	0.39	1.52
**AC_final_uninjured**	0.4339	0.2046	0.42	0.28	0.53	0.12	0.86
**AC_pre_uninjured**	0.4293	0.2180	0.39	0.29	0.57	0.10	0.90
**LARS acute**							
**AC_final**	0.5312	0.2864	0.46	0.33	0.65	0.11	1.63
**AC_pre**	0.9489	0.4171	0.91	0.6125	1.2175	0.34	2.75
**AC_final_WB**	0.6520	0.3022	0.55	0.44	0.77	0.25	1.82
**AC_pre_WB**	1.1862	0.4408	1.115	0.8875	1.485	0.40	2.88
**AC_final_uninjured**	0.4590	0.1718	0.44	0.33	0.55	0.16	1.22
**AC_pre_uninjured**	0.4981	0.1921	0.49	0.36	0.5975	0.19	1.49
**LARS chronic**							
**AC_final**	0.6595	0.4078	0.49	0.38	0.97	0.11	1.69
**AC_pre**	1.1362	0.5061	1.045	0.81	1.32	0.41	2.75
**AC_final_WB**	0.8145	0.4171	0.685	0.49	1.0675	0.36	1.88
**AC_pre_WB**	1.3652	0.4728	1.17	1.0925	1.5675	0.69	2.88
**AC_final_uninjured**	0.4638	0.1470	0.445	0.345	0.5575	0.23	0.99
**AC_pre_uninjured**	0.4481	0.1223	0.425	0.36	0.5575	0.20	0.69
**TightRope^®^**							
**AC_final**	0.7289	0.4853	0.525	0.44	0.955	0.26	2.77
**AC_pre**	1.4489	0.6159	1.315	1.065	1.74	0.34	3.27
**AC_final_WB**	0.8641	0.5276	0.675	0.535	1.085	0.30	2.92
**AC_pre_WB**	1.4658	0.5612	1.35	1.14	1.70	0.45	2.89
**AC_final_uninjured**	0.5417	0.2543	0.49	0.38	0.61	0.10	1.41
**AC_pre_uninjured**	0.5212	0.2570	0.48	0.31	0.63	0.10	1.30

SD: Standard Deviation; Q: Quartile; Min: Minimum; Max: Maximum; AC: acromioclavicular; WB: Weight Bearing; LARS: Ligament Augmentation Reconstruction System.

**Table 5:** Comparison of pre- to postoperative differences (Δ) in AC- and CC-distances between injured and uninjured sides within each cohort.

Measurement (cm)	Injured (Mean ± SD)	Uninjured (Mean ± SD)	*p*-Value
**Bosworth**			
**ΔAC**	0.2616 ± 0.3413	0.0614 ± 0.2693	*p* = 0.0182
**ΔCC**	0.5086 ± 0.5557	0.1377 ± 0.4594	*p* = 0.0087
**LARS acute**			
**ΔAC**	0.4323 ± 0.4114	0.0491 ± 0.1592	*p* < 0.0001
**ΔCC**	0.5919 ± 0.5004	0.0314 ± 0.1814	*p* < 0.0001
**LARS chronic**			
**ΔAC**	0.4767 ± 0.3779	0.0157 ± 0.1215	*p* < 0.0001
**ΔCC**	0.2752 ± 0.5128	0.0069 ± 0.1290	*p* = 0.0015
**TightRope^®^**			
**ΔAC**	0.8403 ± 0.6715	0.0200 ± 0.1498	*p* < 0.0001
**ΔCC**	0.7357 ± 0.6088	0.0746 ± 0.2183	*p* < 0.0001

SD: Standard Deviation; AC: acromioclavicular; CC: coracoclavicular; LARS: Ligament Augmentation Reconstruction System.

**Table 6:** Relative and absolute frequencies of postoperative complications across all cohorts.

Complications	Bosworth	LARS Acute	LARS Chronic	TightRope^®^
**clavicular elevation**				
double shaft width	6.9% (*n* = 2)	0%	0%	0%
full shaft width	20.69% (*n* = 6)	10.00% (*n* = 11)	26.19% (*n* = 11)	9.23% (*n* = 6)
half shaft width	41.38% (*n* = 12)	29.09% (*n* = 32)	21.43% (*n* = 9)	20.00% (*n* = 13)
none	31.03% (*n* = 9)	60.91% (*n* = 67)	52.38% (*n* = 22)	70.77% (*n* = 46)
**AC joint osteoarthritis**				
yes	17.24% (*n* = 5)	8.18% (*n* = 9)	7.14% (*n* = 3)	3.08% (*n* = 2)
no	82.76% (*n* = 24)	91.82% (*n* = 101)	92.86% (*n* = 39)	96.92% (*n* = 63)
**CC ligament ossification**				
yes	37.93% (*n* = 11)	21.82% (*n* = 24)	19.05% (*n* = 8)	7.69% (*n* = 5)
no	62.07% (*n* = 18)	78.18% (*n* = 86)	80.95% (*n* = 34)	92.31% (*n* = 60)

LARS: Ligament Augmentation Reconstruction System; AC: acromioclavicular; CC: coracoclavicular.

## Data Availability

The data that support the findings of this study are available on request from the corresponding author, D.P. The data are not publicly available due to restrictions containing information that could compromise the privacy of research participants.
